# Supplementary material for: Rapid turnover of life-cycle-related genes in the brown algae
Source: Genome Biol. 2019 Feb 14;20:35. doi: 10.1186/s13059-019-1630-6 (PMC6374913; doi:10.1186/s13059-019-1630-6)
Supplement: Supplementary file 3 — Protein alignments, corresponding Gblocks html file and CDS sequences for the lineage conserved gametophyte-biased genes. (ZIP 2109 kb) [file 13059_2019_1630_MOESM3_ESM.zip › Con_GA_alignments/Ec-08_004960.aa_ali.fasta-gb.htm]

Ec-08\_004960.aa.fasta.trans.aa\_ali.fasta


## Gblocks 0.91b Results

Processed file: **Ec-08\_004960.aa.fasta.trans.aa\_ali.fasta**  
Number of sequences: **2**  
Alignment assumed to be: **Protein**  
New number of positions: **947** (selected positions are underlined in blue)

```
                         10        20        30        40        50        60
                 =========+=========+=========+=========+=========+=========+
Ec-08_004960     ------------------------------MENARADECTALGEGSARYDGTKVAATQAF
MSTRG.33353.1    SVDKSTRAASTKQLKVNSLSAGCQTDLLPDRDAAAEEARDTMADTQHATERTALSGGSAR
                                                                    #########


                         70        80        90       100       110       120
                 =========+=========+=========+=========+=========+=========+
Ec-08_004960     SPDTSAAPPAAALGMRSNRSSLSRLAVAVLSISAMFLVGVLLSSTANIHSTR----ENSS
MSTRG.33353.1    YDDRAPVAQTTGLGLRSNRRSLSQLALAVIIISAIFLTGVLLSSAVHFSTKRGSAGSSSR
                 ###         ########################################      ##


                        130       140       150       160       170       180
                 =========+=========+=========+=========+=========+=========+
Ec-08_004960     GRDSSSNSSSEAALNMLDSSAAA-----DG-KIVVPAMQPFSTVDPADAHCPHMDRPEDT
MSTRG.33353.1    GSSSSAQEGSLVEDTARDGRAAASLGPADHEKTIIPAMQPFSTVDPADAFCPHMDRPEDS
                 #######################        #############################


                        190       200       210       220       230       240
                 =========+=========+=========+=========+=========+=========+
Ec-08_004960     WPTSAWGALANASVSLHTSLPTNAWWENIVLGFPSQETRSNNIVVIPYTFDVAGDTAGLR
MSTRG.33353.1    WPTKAWGVLANASVNLHTSLPTNAWWENIVLGFPSQEAKENNIVVVPYTFDVAGDTAGLR
                 ############################################################


                        250       260       270       280       290       300
                 =========+=========+=========+=========+=========+=========+
Ec-08_004960     VHFPQVVASDLIVQTTFDWRHALQLGCVEKVTDHYITQPGPLSVTLNWDVAAGVTSA---
MSTRG.33353.1    VHFPQVMATDLIVQTTFDWRHALQLGCVERVSDHYITQPGPLSVTLNWDLAAGIQGAAAG
                 #########################################################   


                        310       320       330       340       350       360
                 =========+=========+=========+=========+=========+=========+
Ec-08_004960     -GAPAMSVPAVRGSPYATMEYRGVRPVISARQEVTQLVVDGERLSLSSGCENGTSVHVER
MSTRG.33353.1    GGGAAMSVPAVRGSPYATMEYRGVRPVVSARQEVTQLIVDGELLPLSSGCKNGTSLHVER
                  ###########################################################


                        370       380       390       400       410       420
                 =========+=========+=========+=========+=========+=========+
Ec-08_004960     EVEIIFSGGAVSSDDTWLVFVSHPAEFLCSSWFAMERGEPPTPGAVGAWQSMPRFRLEAV
MSTRG.33353.1    EVEIIFSGGAVSSDDTWLVFVSQPTDFLCSLWFAMERGDPPTPGALGAWHSMPRFRLEAV
                 ############################################################


                        430       440       450       460       470       480
                 =========+=========+=========+=========+=========+=========+
Ec-08_004960     RAVSEEEGGVVRAAMVNTCTSGTSNRCTKPGEPDDREDYAKLLRAHAEVYPTGQARVSYS
MSTRG.33353.1    EPASEEEGAVVRAAVLNTCTSGTSDRCEKPGEPDDRTEYARLLRAHAEVYPTGQARVSYA
                 ############################################################


                        490       500       510       520       530       540
                 =========+=========+=========+=========+=========+=========+
Ec-08_004960     SSSLDWIEDALQGDVAQTADTARLEEDTQNSAMTAPASAGASPKP-RAESEGDG--AEEE
MSTRG.33353.1    SSTLDWIEDALEAAEAVAIDRDSEGTAAAGASTGDVKSAELDTEDDKASDSGDAGLRDEE
                 ####################                                      ##


                        550       560       570       580       590       600
                 =========+=========+=========+=========+=========+=========+
Ec-08_004960     GSLRFLWAPKRMDGKPVVAVDPAAAVAAAAAAAGGDDGGSDSGESDSSAGDSDSADVGAD
MSTRG.33353.1    GTLRFLWAPRRMDGKPIV--DSTAPMSAAVAAAAGDDSGD--GDTDSSGDGDDGSSRDDG
                 ##################  ###################   ##################


                        610       620       630       640       650       660
                 =========+=========+=========+=========+=========+=========+
Ec-08_004960     SPPLIMFALLHHRQAFTAADGLTDIGTPSLHGQAEVAIGNEWSMKVALPPVSFGVGRPVR
MSTRG.33353.1    SVPLIMFALLHHRQAFTDADGMTGVGTPTLHGQANLAIGDEWSMKVALPPVSFGVGRPVR
                 ############################################################


                        670       680       690       700       710       720
                 =========+=========+=========+=========+=========+=========+
Ec-08_004960     EEMVADVNEALDEDVKYEMPKNYLRGEGDTYFSGKMLAKMGRIALIAEEMGRPEDARMVA
MSTRG.33353.1    EEMVGAVNDALDEDVKYEMPGNYLRGDGDTYFSGKMLAKMGRIALIAEEMGRLEDARTVA
                 ############################################################


                        730       740       750       760       770       780
                 =========+=========+=========+=========+=========+=========+
Ec-08_004960     ARLAEASQVWLNGTAGSPLLYDFRWGGVVTCGCAYKKDHGCTNGIGPYQCPGLSDPGMNF
MSTRG.33353.1    ARLAEASQVWLNGTAGSPLIYDFRWGGVVTCGCAYKKGHGCTNGIGPHECPSLSDPGMNF
                 ############################################################


                        790       800       810       820       830       840
                 =========+=========+=========+=========+=========+=========+
Ec-08_004960     GLGFYNDHHFHFGYHIFAAAIVSKFFPDWGIKHHEAVTLLIRDIANPSSSDPFFPVFRHK
MSTRG.33353.1    GLGFYNDHHFHFGYHIFAAAVAAKFYPEWGVKHHEAVTLLIRDIANPSSSDPFFPVFRHK
                 ############################################################


                        850       860       870       880       890       900
                 =========+=========+=========+=========+=========+=========+
Ec-08_004960     DWFLGSSWALGIPTLGGVPYMNGRNQESSSEAVNAYYAVALYGHVMAQVFSSAGDPAKTQ
MSTRG.33353.1    DWYLGSSWALGIPTLGGVPYMNGRNQESSSEAVNAYYGVALYGHVMAQVFSTAGDPAKAQ
                 ############################################################


                        910       920       930       940       950       960
                 =========+=========+=========+=========+=========+=========+
Ec-08_004960     TASLIRDTGRQLLATEVQSAQIYWQVANAGTPDLPRVYPEAYRPHVVGMLWSTLAQMQTW
MSTRG.33353.1    TASLIRDTGRQLLATEVQAAQIYWQVEDPGTPDMPRVYPEAYQPHVVGMLWSTLAQMQTW
                 ############################################################


                        970       980       990      1000      1010      1020
                 =========+=========+=========+=========+=========+=========+
Ec-08_004960     FGAEAWKVYGIQMLPITGVSEQLLDPRWVQQMLPSFEDSCLYDSNPIKACVVEGWSMLVH
MSTRG.33353.1    FGAEAWKVYGIQMLPITAASEQLLDPRWVQQMLPSFEDSCLYDNNPIKACVVGGWSMLVH
                 ############################################################


                       1030      1040      1050      1060      1070      1080
                 =========+=========+=========+=========+=========+=========+
Ec-08_004960     AGQAITGRWEDGRDGIMALPDDVFETAGGNGHSRTSMLWFVANRPPPPPP-S-------G
MSTRG.33353.1    AGQAITGRWEDGRDGILALPDGAFETAGGNGHSRTSMLWFVANRPPAPAVIHGDSDSESD
                 ################################################            


                 
                 =====
Ec-08_004960     DDDSY
MSTRG.33353.1    SNDSR
```

```
Parameters used
Minimum Number Of Sequences For A Conserved Position: 2
Minimum Number Of Sequences For A Flanking Position: 2
Maximum Number Of Contiguous Nonconserved Positions: 8
Minimum Length Of A Block: 10
Allowed Gap Positions: None
Use Similarity Matrices: Yes
```

```
Flank positions of the 8 selected block(s)
Flanks: [52  63]  [73  112]  [119  143]  [152  297]  [302  500]  [539  558]  [561  579]  [583  1068]  

New number of positions in Ec-08_004960.aa.fasta.trans.aa_ali.fasta-gb:  947  (87% of the original 1085 positions)
```
